# Who’s Getting a Head Start? Mesocephalic Dogs in Still Images Are Attributed More Positively Valenced Emotions Than Dogs of Other Cephalic Index Groups

**DOI:** 10.3390/ani12010049

**Published:** 2021-12-27

**Authors:** Bonita L. Brincat, Paul D. McGreevy, Verity A. Bowell, Rowena M. A. Packer

**Affiliations:** 1 Royal (Dick) School of Veterinary Studies, University of Edinburgh, Edinburgh EH25 9RG, UK; verity.bowell@ed.ac.uk; 2 Faculty of Science Agriculture and Law, School of Environmental and Rural Science, University of New England, Armidale, NSW 2351, Australia; pmcgree2@une.edu.au; 3 Royal Veterinary College, Hawkshead Lane, Hatfield AL9 7TA, UK; rpacker@rvc.ac.uk

**Keywords:** brachycephaly, welfare, emotional attribution, dogmanship, ownership, canine

## Abstract

**Simple Summary:**

Elements of a dog’s appearance in still images affect how positively human observers interpret that individual’s personality. Given that this may influence caregiving and other aspects of dog ownership, it is important to examine this phenomenon to protect dog welfare. Recently, the popularity of brachycephalic (short-muzzled) dogs has sharply risen and with it the need to assess whether this conformation affects the way in which human observers assign emotional attributes to dogs. The current study aimed to investigate whether cephalic index, a measure to quantify how long and wide a skull is, is related to how both dog owners and non-dog owning adults in the U.K. attribute emotions to still images of dogs, and in the case of dog owners, to their own dogs. Responses were received from 2451 participants. Images of breeds with less extreme skull shapes were most frequently assigned the strongest positive emotional attributions, and the inverse effect was found in more extreme skull shape categories. Results imply that the head shape of dogs may predispose humans to label those dogs with certain emotions, which could impact their behaviour towards those dogs and thus, the dogs’ welfare. These findings should prompt further investigation of morphological influences on dog–owner relationships and dog welfare.

**Abstract:**

Assumptions about dogs’ personality are influenced by their appearance, which may lead to differences in ownership styles and subsequent canine welfare. The influence of canine appearance on observers’ emotion attributions to dogs remains largely unexplored. This study investigated whether canine head shape is related to how both dog owners and non-dog owning adults in the U.K. attribute emotions to still images of dogs, and in the case of dog owners, to their own dogs. Attachment, respondent personality and dog trainability were assessed as potential influences on emotional attribution in owners. Overall, 2451 participant responses were received. Still images of mesocephalic dogs were attributed primary and positively valenced emotion with more strength and frequency than other groups. Mesocephalic images were also attributed negatively valenced emotions less frequently and with less strength than other groups. Apart from empathy, no significant differences were found in emotional attribution to owned dogs of different head shapes; however, human personality influenced attribution of emotions to owned dogs. The finding that some dogs are attributed emotions more readily based on their appearance alone has applied importance, given, for example, the potential for misattribution of positive emotions to dogs in negative emotional states, and potential prejudice against dogs considered in negative emotional states.

## 1. Introduction

It is estimated that more than 9.8 million dogs resided in the United Kingdom (U.K.) in 2019 [1], with owners often referring to their dog as a “family member” [2]. Owners consistently report primary and complex secondary emotions in their cats and dogs [3,4], albeit secondary emotions are reported less frequently in cats than in dogs [4,5]. This follows a widespread belief that dogs experience complex secondary emotions such as guilt [6], jealousy [7], and spite [8]. Though there is general scientific agreement that mammals experience basic emotions [9], there is scant evidence that companion animals experience an emotional range akin to humans [6,10,11]. Secondary emotions require self-consciousness [12,13]; thus, they are considered exclusive to primates [11], although at least one type of jealousy without self-consciousness in dogs has been proposed [7,14]. An owner’s attribution of secondary emotions to dogs may create risks to canine welfare. Lindsay [15] and Rajecki et al. [16] suggest that owners use punishments when problem behaviours are ascribed to internal motivations, e.g., an internal emotional drive, such as spite. As punishment-based training methods are implicated in poor canine welfare [17,18,19], understanding factors linked to canine emotional attributions is therefore potentially important in identifying dogs at risk of poor welfare.

Two biological mechanisms have been proposed to explain some core aspects of owner–dog relationships. The theory of “kindenschema” [20], describing the Baby Schema Effect (BSE), suggests parents are primed for caregiving by being triggered by “infantile” features such as large eyes that are present in human infants, and young of other species including dogs [21]. Similarly, the theory of attachment [22,23] identifies behavioural manifestations of attachment bonds between caregivers and human infants, and potentially between dogs and owners [24]. These theories underpin the development and progression of parental caregiving and eventual attachment bonding and are thought to contribute strongly to the dog–owner bond. These mechanisms, particularly BSE, may influence the way humans think and feel about dogs with certain conformational traits. Visual traits linked to juvenility can influence human ratings of personality traits in dogs. Fratkin and Baker [25] found that dogs with floppy ears or light coats were rated higher on agreeableness and emotional stability than those with erect ears or dark coats, respectively. Thorn et al. [26] investigated the perceived “cuteness” of still canine images and the behavioural characteristics respondents attributed to them. Although cuteness is not an extensively researched feature in dogs, it has been linked to BSE conformation [27], likely through similar mechanisms. Dogs rated as “cuter” were also rated as more amicable than those rated “less cute”, suggesting that visual familiarity and “cuter” appearance are important to the human–dog bond, as well as owner perceptions of dog personality [21].

There is evidence that BSE in human infants initiates elements of caregiving in adults, such as increased visual vigilance and fine motor dexterity, that underpin provision of care to sensitive infants [28]. One study [28] reported the same results when human participants were exposed to juvenile canine images, suggesting that the BSE effect extends to dogs. As similar studies use differing methods, such as altering features, to fit BSE to a greater or lesser extent [29] and use adult versus juvenile faces to represent BSE [28], their results defy extensive comparison. However, findings between studies are generally consistent, suggesting that BSE in dogs can have a profound influence on the behaviour of observing humans.

The cephalic index (CI) is a measure of head shape—the ratio of the width of the skull divided by its length. Numeric CI scores are categorised as brachycephalic (BC), mesocephalic (MC) and dolichocephalic (DC) by some authors [30], describing whether the dog has a comparatively high (BC), low (DC) or mid-range CI score (MC). Concerns for the health and welfare of BC types have grown among the veterinary community [31], as dogs and cats with extreme brachycephaly are often born with or develop health disorders inherently associated with their skull [32,33,34,35]. Paradoxically, these extreme morphologies may contribute to the current popularity. Farnworth et al. [36] found that MC cats were preferred over BC and DC feline faces. However, preference can reflect a respondent’s familiarity with certain morphotypes [26] and so may arise as a result of BC and DC cats being rarer than their MC counterparts in some countries, e.g., the U.K. [37]. While this may be due to low levels of BC or DC cats in some pet cat populations, this is less likely for dogs because ownership of BC and DC dog breeds is extremely common at present [31]. Indeed, recent studies indicate that the appearance of brachycephalic breeds is the strongest influence on owner choice of these breeds, with canine appearance comparatively more important for owners of brachycephalic than non-brachycephalic breeds [38,39].

Given that positive emotional attribution is linked to canine appearance and BSE, dogs across the CI scale may be attributed emotions differently, such that some dogs with certain facial configurations may be at risk of less frequent attribution of positive temperament traits, such as amicability. Because previous studies have shown that humans can assign dogs negative, secondary emotions such as guilt [6], research investigating associations between canine appearance and emotional attribution is needed to identify conformations at risk of emotional misattribution.

The current study primarily aimed to investigate whether CI score is related to how both dog owners and non-dog owning adults in the U.K. attribute emotions to still images of dogs, and in the case of dog owners, to their own dogs.

The study tested three hypotheses:

- The frequency and strength of emotional attribution differs significantly between CI groups of still images of dogs, with higher CI scores associated with more frequent and stronger attribution of positive and secondary emotions.
- The frequency of emotion attribution differs significantly between CI groups of respondents’ owned dogs, with higher CI scores associated with more frequent attribution of positive and secondary emotions.
- The frequency of secondary and individual emotion attribution differs significantly between CI groups of still images of dogs and is associated with the CI group of a respondents’ own dog.

The secondary aim of this study was to investigate how facets of the human–canine relationship may impact emotion attribution, including dog–owner attachment, owner personality and owner perceptions of canine behaviour.

## 2. Materials and Methods

### 2.1. Recruitment and Sampling

A questionnaire surveyed participants for seven weeks between 20 January 2020 and 9 March 2020 using snowball sampling [40] and convenience sampling to recruit participants. The social media platform Facebook (https://www.facebook.com, accessed on 3 July 2019) was the primary distribution method, with survey details and a link to the study posted to 47 U.K. Facebook “groups”, subject to group administrator permission. Groups based on a canine topic were selected to increase the likelihood of engagement, and to recruit dog owners specifically for hypotheses two and three. The survey link was also disseminated through the newsletter of a large U.K. dog charity, and through personal correspondence with those known to the primary researcher, a canine behaviour professional in the U.K.

### 2.2. Cephalic Index Data

Archived CI data from McGreevy et al. [30] were obtained with permission from the corresponding author. These data were taken from 80 breeds and drawn from physical measurements of pedigree dogs’ heads at dog shows in Australia, with at least six male and female registered individuals of each breed. A mean CI score for each breed was calculated by combining CI scores for individuals from the same breed and calculating the mean of the male and female CI scores for each breed. To assign breeds to CI groups, mean scores for each breed were calculated. In the absence of consensus in the literature of distinctions defining specific skull conformational categories based on CI, the data were divided into terciles. Breeds in the lowest tercile of the dataset of mean CI scores (36.85–59.52) were classified as dolichocephalic (DC), those in the mid-tercile (59.63–80.59) were classified as mesocephalic (MC) and those in the highest tercile (80.60–101.59) were classified as brachycephalic (BC). After five breeds were removed (four due to lack of popularity in the U.K. and one due to researcher error), CI data for 75 breeds were available for use in the final study.

### 2.3. Dog Images to Assess Viewer Emotional Attribution

**Figure 1:**
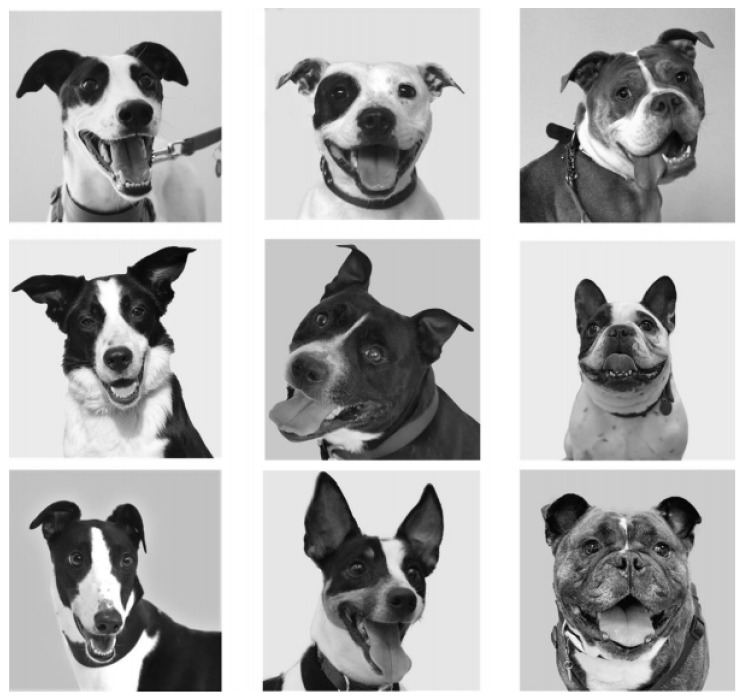
Final matched images of dogs of varying CI groups. Columns show images presenting, from left to right, DC, MC and BC groups.

The images taken from a U.K. rescue’s website were categorised as within CI groups described above based on the breed designation they had been given by the rescue organisation. Respondents’ dogs were also categorised into the same CI groups if they reported ownership of a breed measured within McGreevy’s study [30]. All respondents were shown nine images of dogs used with permission from one large U.K. animal rescue rehoming website and split into three CI groups using the data and methods described previously. Images were selected on the shelter’s breed designations of dogs involved (DC dogs: 2 × Greyhounds, 1 × Border Collie. MC dogs: 2 × Staffordshire Bull Terriers, 1 × Jack Russell Terrier. BC dogs: 2 × British Bulldogs, 1 × French Bulldog). Three images of each CI category (nine images in total) were selected in order to include a variety of breeds. This minimised the risk of respondents attributing emotions to CI groups based on potential breed preconceptions, which may otherwise skew results. Images were limited to three per CI group to limit survey length and respondent fatigue whilst completing the survey. Due to the limited number of images available, images selected for use in the survey were matched primarily for features that may otherwise influence respondents’ interpretation of emotion. No specific facial expressions were intentionally selected for the current study, though due to their original source and purpose the photographs, i.e., promoting individual dogs for rehoming, they may have been selected by the rehoming organisation to exclude facial expressions perceived as projecting a negative emotional state. Images were presented in greyscale, resized to 500 × 500 px and matched on features which may otherwise have influenced respondents’ attributions: blank background, camera-facing headshot, erect or semi-erect ears, sclera showing, mouth open with visible teeth and tongue, facial patches with contrasting light and dark colouring, a white central blaze or stripe and wearing a collar (Figure 1).

Images were presented to respondents in the same order (British Bulldog, Staffordshire Bull Terrier, Greyhound, Jack Russell Terrier, British Bulldog, Greyhound, Staffordshire Bull Terrier, Border Collie, French Bulldog). This order was devised to avoid framing effect biases. No two dogs of the same CI category were presented consecutively, and all CI groups were represented in the first three images seen by participants.

Respondents were asked to rate on a 5-point Likert scale (strongly agree to strongly disagree) their agreement with the attribution of the eight emotions listed above for each image. These data were also transformed into a binary variable: responses rating “agree” or ”strongly agree” were recorded as an attribution of the emotion to the image (1); conversely, responses rating ”strongly disagree”, “disagree” or “neither agree nor disagree” were recorded as the respondent not having attributed the emotion to the image (0). Thus, for each image, both binary attribution and strength of attribution were recorded for each emotion and analysed separately.

### 2.4. Survey Design

The questionnaire was hosted by JISC (http://onlinesurveys.ac.uk, Accessed on 3 July 2019). The first page outlined a consent statement that respondents had to agree to prior to participation. Responses collected no personal identifying data and were anonymised, minimising the likelihood of response bias as well as adhering to U.K. regulations and ethical best practices.

#### 2.4.1. Demographic Information

Demographic information was collected from all respondents about their age, gender, level of education and professional experience with animals. Data were collected on whether the participant owned or was responsible for a dog to determine whether they were presented with questions related to hypothesis two and three of aim one or aim two questions.

#### 2.4.2. Dog Ownership Information

Respondents identifying as dog owners were asked if they owned one or more dogs; those with more than one dog were asked to select the dog whose first name came first alphabetically to avoid bias. Participants of recognised breeds were asked to report their dog’s breed. If the dog was of a breed listed in the CI data collected from McGreevy et al. [30], it was assigned to one of the three head shape categories as outlined above. Respondents owning mixed-breed dogs and whose pedigree breed did not have available CI data were excluded from statistical analyses on owner-reported data regarding their owned dog.

To allow description of the study population, owners further reported their ownership and breed experience and their study dog’s age, sex, neuter status, length of time owned, source and purpose of acquisition.

#### 2.4.3. Attribution of Emotions to Owned Dogs

Participants answered using a yes/no response whether they believed their dog could experience the primary emotions of joy, affection, sadness and fear [41] and secondary emotions of embarrassment, empathy, compassion and spite [42]. Emotions were categorised into positive (joy, affection, compassion, empathy) or negative (sadness, fear, embarrassment, empathy) emotional valence (Table 1).

#### 2.4.4. Lexington Attachment to Pets Scale

Dog-owning respondents completed the Lexington Attachment to Pets Scale (LAPS) [43] as a measure of attachment to their dogs. LAPS is a validated, 23-item scale designed to measure the strength of owner–pet attachment using 5-point Likert response options and two reverse-scored items. Johnson et al. [43] reported a Cronbach’s alpha value of 0.94, demonstrating excellent internal consistency and rendering it a useful tool for the current study.

#### 2.4.5. Trainability

The trainability subscale of the Canine Behavioural Assessment and Research Questionnaire (C-BARQ) [44] was completed by dog-owning respondents. This is an 8-item subscale designed to measure the reported trainability of respondents’ dogs using a 7-point Likert scale with two items reverse-scored. For the current study, a “not applicable” response option was added for those with no experience of the behaviour described. For analysis, responses using “not applicable” selections were treated as missing values, with average scores calculated from the remaining responses without missing values.

#### 2.4.6. Ten-Item Personality Inventory

All respondents completed the Ten-Item Personality Inventory (TIPI), a scale validated in humans, measuring the “Big Five” personality dimensions: extraversion, openness to experience, conscientiousness, emotional stability and agreeableness [45]. Each subscale consisted of two items using a 7-point Likert scale, with one question reverse-scored. This abridged version of the well-known psychometric test was selected to minimise the risk of respondent fatigue.

#### 2.4.7. Self-Help Resources

Upon completion of the survey, all participants were signposted to free self-help resources for their benefit in the unlikely event that over the course of the questionnaire, emotional distress had been experienced. These resources were available at no charge and were sourced from established charities and organisations concerned with improving human mental health or canine behaviour and welfare.

### 2.5. Statistical Analysis

Survey data were exported into the Statistical Package for Social Sciences version 24.0 (IBM SPSS Statistics for Windows, Version 24.0. Armonk, NY, USA) for statistical analysis. Descriptive statistics (frequencies and percentages) were calculated for demographic and dog-based variables.

Differences in scores between the three CI groups of dog images (BC, MC, DC) were analysed using one-way ANOVA and independent samples Kruskal-Wallis (ISKW) tests depending on normality of data distribution, assessed using histograms. Post-hoc tests identified significant differences between the three groups using Fisher’s least significant difference test for ANOVA analyses, and pairwise comparisons using Dunn’s post-hoc tests for ISKW tests.

For categorical data, the Chi-squared test was used to identify associations between variables (e.g., CI groups vs. binary attribution (Y/N) of an emotion to their own dog). Spearman’s rank correlation coefficient was used to identify correlations between two continuous variables and identify which direction relationships were in.

Total scores and means were calculated for the three validated scales (and their sub-scales, where applicable) that were administered (LAPS, TIPI and C-BARQ) in accordance with their published methods. These variables were then analysed against individual EA (Emotional Attribution) to both still images of dogs and, where relevant, their own dogs using Spearman’s rank to detect significant correlations.

Descriptive data were calculated from individual question items to create total scores for EA categories for the dog images and EA to respondents’ own dogs; for example, scores for all four primary emotions toward still images were grouped to create an overall score for primary attribution to still images. Likert-scale data on strength of EA were treated as continuous scale data and means were calculated. Additionally, EA variables (attributed or not: 1/0) were calculated by grouping emotion responses to images and owned dogs into positive or negative emotions, primary or secondary emotions and total emotions attributed categories.

After analysis, *p* values were adjusted using the false discovery rate to correct for multiple comparisons. All results where *p* < 0.05 were considered significant.

### 2.6. Ethical Approval

Ethics approval was received for this research from Edinburgh Human Ethical Review Committee (HERC 447-19). For usage of secondary data from McGreevy et al. [30], approval from the Edinburgh Veterinary Ethical Review Committee was obtained (VERC 151.19). Participants in this study met selection criterion of adults (18 or over) residing in the U.K.

## 3. Results

### 3.1. Responses and Descriptive Statistics

In total, 2451 responses were obtained. Six responses were removed due to missing values, leaving 2445 valid responses. Of all participants, 90% were female (*n* = 2211), 9% were male (*n* = 220) and the remaining 1% identified as “other” or preferred not to disclose. Around one-fifth of owners (22%, *n* = 530) had professional experience with animals. The majority (*n* = 2080, 84%) were dog owners, with *n* = 385 reporting that they had previously owned or been responsible for a dog. Of dog owners, 1451 (1451/2060, 59%) owned dogs of a registered breed, and 1174 (1174/1451, 81%) owned dogs of a breed recorded in the study by McGreevy et al. (2013) for which approximate CI scores were available and CI group had been estimated. Of these 1174 dogs, 614 were DC (52%), 425 were MC (37%) and 135 were BC (12%).

### 3.2. Impacts of CI Group on Emotional Attributions of Nine Dog Images

#### 3.2.1. Total Attributions to Images and Individual Emotions

**Figure 2:**
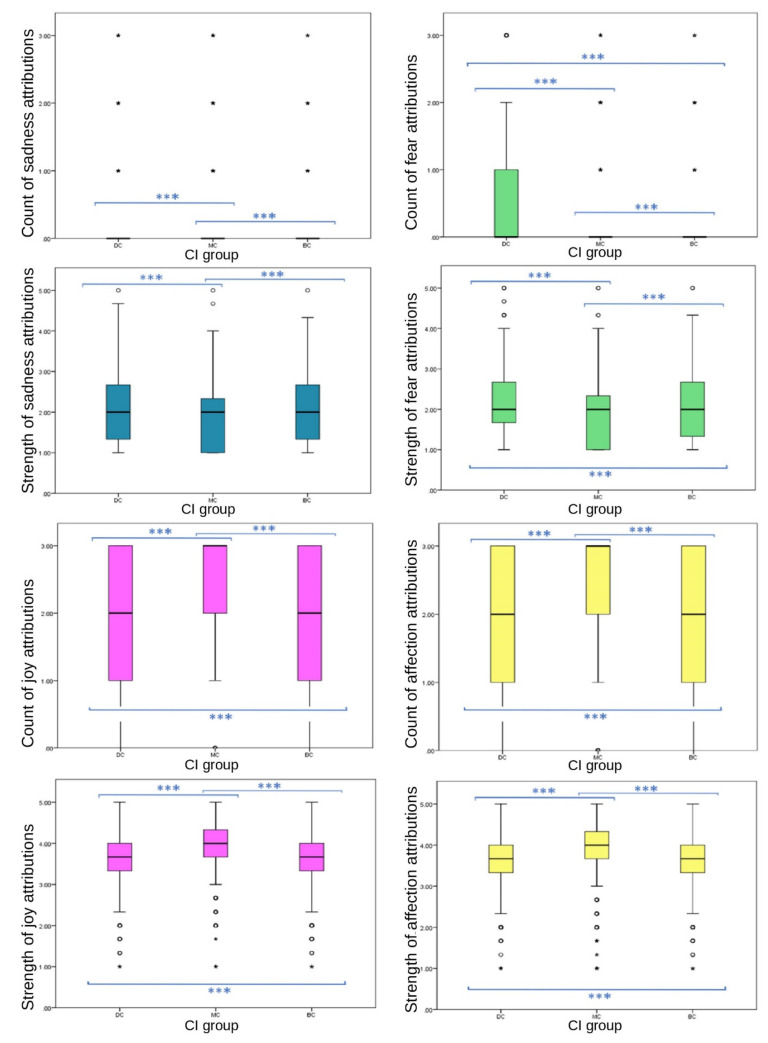
Box plots describing mean scores of individual primary emotion attribution strengths and frequencies between CI groups of dog images. (*** demotes statistically significant differences. ◦ denotes outliers).

**Figure 3:**
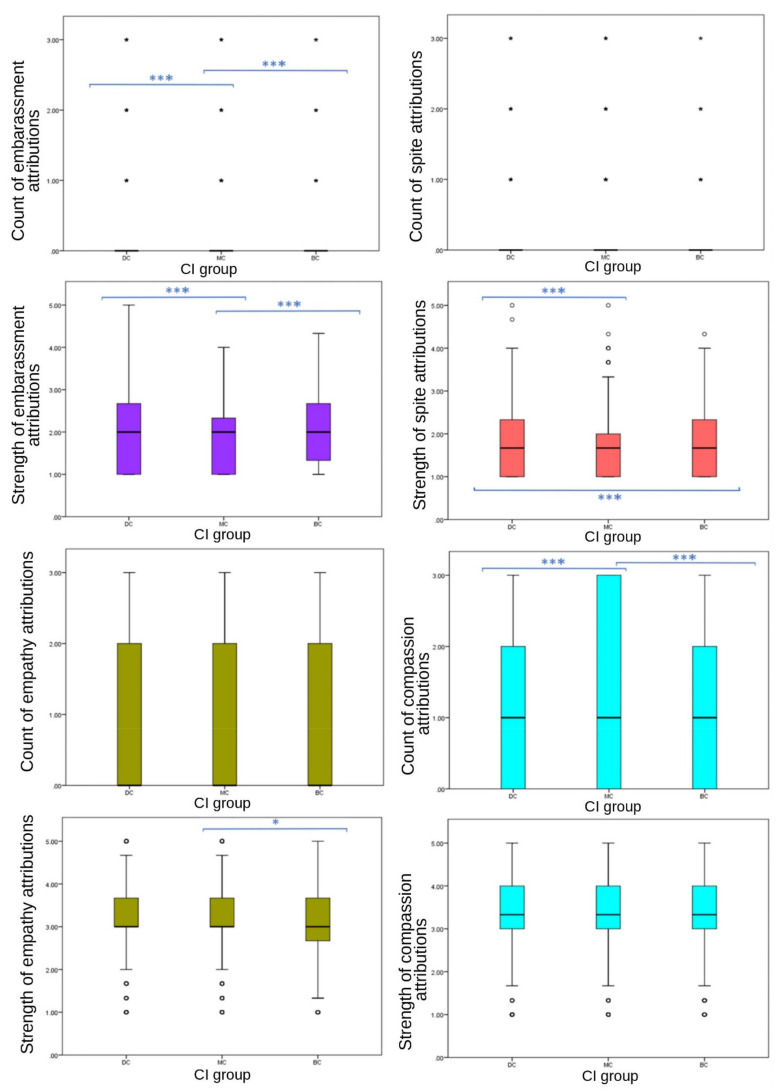
Box plots describing mean scores of individual secondary emotion frequencies and strengths between CI groups of dog images. (*** demotes statistically significant differences. ◦ denotes outliers.)

Strong significant differences between CI groups in EA were observed across strengths and frequencies of emotions attributed to images. Significant differences were found between CI groups for the total number of emotions attributed to still images; MC images received the highest frequency of EA overall (one-way ANOVA (F (27,332) = 34.088), *p* ≤ 0.001). Significant differences were found between CI groups in frequencies of the attribution of joy (one-way ANOVA (F (27,332) = 320.779), *p* ≤ 0.001), sadness (ISKW, X^2^ = 192.109, *p* ≤ 0.001), fear (ISKW, X^2^ = 184.207, *p* ≤ 0.001), affection (one-way ANOVA, (F ^(27,332)^ = 79.554), *p* ≤ 0.001), compassion (ISKW, X^2^ = 13.331, *p* ≤ 0.001) and embarrassment (ISKW, X^2^ = 78.944, *p* ≤ 0.001). No significant differences were found between CI groups of the EA frequency of spite (ISKW, X^2^ = 2.386, *p* = 0.303), strength of compassion (one-way ANOVA, F ^(27,332)^ = 3.351, *p* = 0.071), or the frequency (ISKW, X^2^ = 6.576, *p* = 0.087) or strength (one-way ANOVA, F ^(27,332)^ = 3.152, *p* = 0.100) of empathy. Individual emotions of joy (one-way ANOVA (F ^(27,332)^ = 282.035), *p* ≤ 0.001), affection (one-way ANOVA (F ^(27,332)^ = 62.082), *p* ≤ 0.001), sadness (ISKW, X^2^ = 86.136, *p* ≤ 0.001), fear (one-way ANOVA, F ^(27,332)^ = 88.729) *p* ≤ 0.001), spite (one-way ANOVA, (F ^(27,332)^ = 8.718), *p* ≤ 0.001) and embarrassment EA (one-way ANOVA, (F ^(27,332)^ = 21.138) *p* ≤ 0.001) showed significant differences in strength between CI groups (Figure 2 and Figure 3).

#### 3.2.2. Frequency and Strength of Emotion Attribution to Primary and Secondary Emotions

Primary grouped variables included emotions joy, affection, sadness and fear. Emotions grouped to create the secondary emotion category were spite, embarrassment, compassion and empathy.

Significant differences were found between CI groups in EA of grouped primary emotions, with MC receiving the highest count (one-way ANOVA (F (27,332) = 86.589), *p* ≤ 0.001); however, no significant differences were found between CI groups for grouped secondary emotions (one-way ANOVA, F ^(27,332)^ = 2.893, *p* = 0.055). Grouped primary emotions showed significant differences in strength between CI groups (one-way ANOVA (F (27,332) = 6.589), *p* = 0.004); however, there were no significant differences between CI groups of grouped secondary EA strength (one-way ANOVA, F ^(27,332)^ = 1.796, *p* = 0.166).

All primary emotions reported significant differences in EA between CI groups. Of these, two positive emotions (joy and affection) showed significant differences between CI groups for frequency (joy, *p* ≤ 0.001 between both BC-MC and DC-MC groups, *p* = 0.023 between BC-DC groups; affection, *p* ≤ 0.001 between all CI groups). Of these, MC received significantly higher frequency of EA (joy, mean = 2.48, SD = 0.821; affection, mean = 2.24, SD = 1.034) than BC (joy, mean = 1.91, SD = 1.011; affection, mean = 1.99, SD = 1.075) and DC (joy, mean = 1.85, SD = 1.054; affection, mean = 1.86, SD = 1.129) groups. Of the primary negative emotions, EA of sadness followed an inverse pattern to that of the above primary positive emotions, with MC images receiving significantly lower EA (median = 0, IQR = 0, 90th percentile = 0) than BC (median = 0, IQR = 0, 90th percentile = 1) and DC (median = 0, IQR = 0, 90th percentile = 1) images. Differences were significant at the *p* ≤ 0.001 level between BC-MC and DC-MC groups, while the difference between BC-DC groups was not significant (*p* = 1.00). The frequency of the attribution of fear did not follow this pattern. DC images received significantly more fear EA frequency (median = 0, IQR = 1, 75th percentile = 1) than MC (median = 0, IQR = 0, 75th percentile = 1) and BC (median = 0, IQR = 0, 75th percentile = 1) images. Significant differences were evident among the three groups (*p* ≤ 0.001). DC images received the most attributions. The difference between BC-DC groups for frequency of fear was larger than the other negative emotion variables (MD between groups: DC-MC = −588.412, BC-MC = 237.256, BC-DC = 351.156).

**Figure 4:**
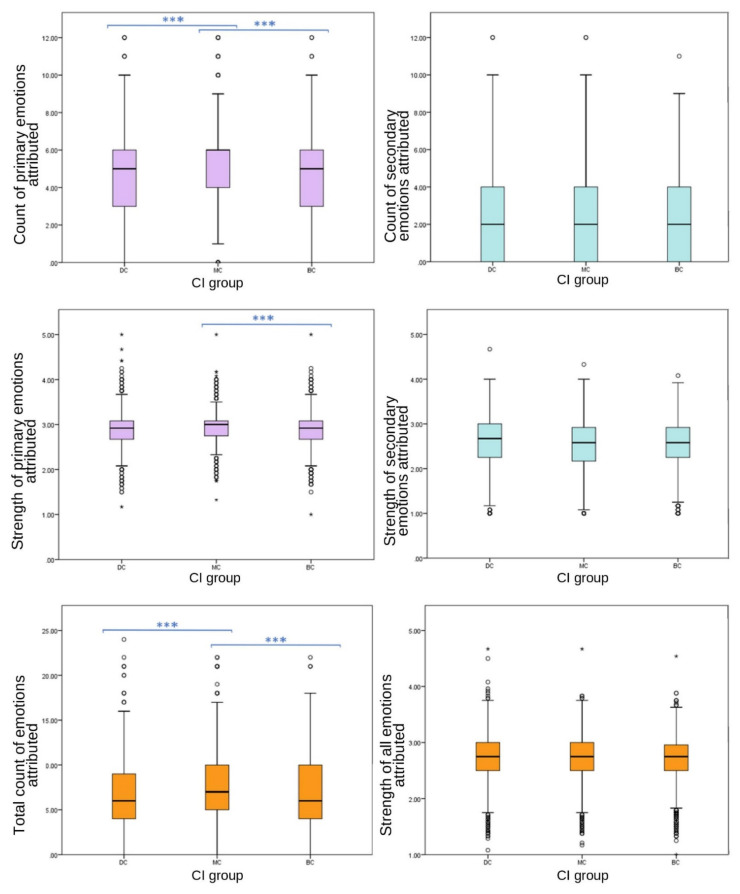
Box plots describing mean scores of grouped primary, secondary and total emotion attribution strength and frequencies between CI groups of dog images. All variables were calculated using four emotions, apart from the total count and strength emotions, which included eight emotions. Strength was measured using a 5-point Likert scale. (*** demotes statistically significant differences. ◦ denotes outliers.)

Results were less consistent for secondary emotions (Figure 4). No significant differences were observed in frequency of spite EA (*p* = 0.422), or either frequency (*p* = 0.087) or strength (*p* = 0.100) of empathy EA between CI groups. Frequency of compassion (*p* ≤ 0.001), and both frequency (*p* ≤ 0.001) and strength (*p* ≤ 0.001) of embarrassment, and the strength of spite (*p* ≤ 0.001) EA showed significant differences between CI groups. Attribution frequency and strength of embarrassment showed a consistent significant difference across CI groups, with MC images receiving significantly fewer attributions (median = 0, IQR = 0, 90th percentile = 0) and weaker attributions (mean = 1.89, SD = 0.749) than BC (frequency: median = 0, IQR = 0, 90th percentile = 1; strength: mean = 2.01, SD = 0.759) and DC (frequency: median = 0, IQR = 0, 90th percentile = 1; strength: mean = 2.03, SD = 0.788) groups. However, DC images received stronger attributions for spite (mean = 1.75, SD = 0.752) than BC (mean = 1.68, SD = 0.696) and MC (mean = 1.67, SD = 716) groups, with no significant difference between MC and BC values. Compassion attributions showed significant differences between CI groups in frequency (*p* ≤ 0.001). Frequency of compassion attributions was significantly different between BC-MC (*p* ≤ 0.020, pairwise comparison test statistic = −154.964) and DC-MC (*p* = 0.002, pairwise comparison test statistic = −198.896) groups, with DC and BC receiving less (both median scores = 1, both IQR = 2, both 75th percentile = 2) compared to MC (median = 1, IQR = 3, 75th percentile = 3). DC images received significantly higher scores than BC and MC groups for fear frequency, strength of fear, strength of spite, negative emotion strength and negative emotion frequency.

#### 3.2.3. Frequency and Strength of Emotion Attribution to Positive and Negative Emotions

Positive grouped variables included joy, compassion, empathy and affection; negative grouped variables included fear, sadness, spite and embarrassment.

The number of respondents attributing positive emotions to images was highest for MC pictures (*n* = 2375, 97%) compared to BC (*n* = 2337, 95%) and DC (*n* = 2294, 94%) groups. The inverse effect was found for negative emotions, with DC images attracting the most respondents attributing negative emotions (*n* = 950, 39%) compared to BC (*n* = 839, 34%) and MC (*n* = 503, 21%) pictures. Among individual EA for positive emotions, MC images received the most attributions (mean = 7.06, SD ± 3.32), DC received the fewest (mean = 5.82, SD ± 3.58) and BC received slightly more than DC (mean = 6.02, SD ± 5.52). Differences between CI groups DC-MC and BC-MC were significant at the *p* ≤ 0.001 level, whilst the difference between DC-BC groups was weaker (*p* = 0.043). Of EA for negative emotions, DC images received the most (median = 0, IQR = 1, 90th percentile = 3), MC received the fewest (median = 0, IQR = 0, 90th percentile = 1) and BC attributions fell between these two groups (median = 0, IQR = 1, 90th percentile = 2). Differences between these CI groups were all significant at the *p* = 0.001 level.

Significant differences were found between CI groups in EA frequency of positive emotions, with MC receiving the highest count (one-way ANOVA, (F (27,332) = 89.379), *p* ≤ 0.001). For negative EA, the inverse effect was found (ISKW, X^2^ = 227.120, *p* ≤ 0.001). MC dogs (median = 0, inter-quartile range (IQR) = 0) received a lower median count for negative emotion attributions compared to DC dogs (median = 0, IQR = 1) and BC dogs (median = 0, IQR = 1).

**Figure 5:**
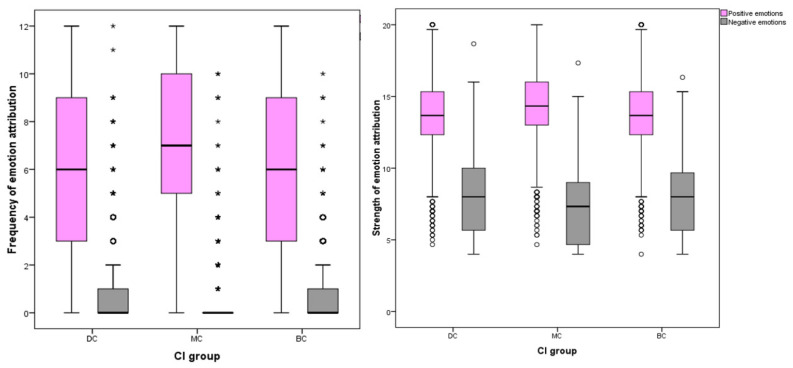
Box plot showing significant differences between positive and negative emotion frequency and strength of emotion attributions to CI groups of dog images. Differences between all groups were significant at the *p* = 0.05 level. Positive grouped variables included joy, compassion, empathy and affection; negative grouped variables included fear, sadness, spite and embarrassment. (* demotes statistically significant differences. ◦ denotes outliers.)

The number of participants attributing negative emotions to dog images was less frequent than those attributing positive EA: sadness (*n* = 786, 32%), fear (*n* = 1011, 41%), embarrassment (*n* = 459, 19%), spite (*n* = 171, 7%), joy (*n* = 2384, 97%), affection (*n* = 2302, 94%), empathy (*n* = 1547, 63%) and compassion (*n* = 1661, 68%). When grouped, significant differences were identified between individual CI groups for both positive and negative EA frequencies (Figure 5).

Similar differences were identified between cephalic groups in the strength of EA. For positive EA, images of MC dogs received stronger attributions than BC or DC images, and the inverse effect was found for negative emotions. Both positive (one-way ANOVA, F ^(27,332)^ = 59.254) *p* ≤ 0.001) and negative EA (one-way ANOVA, F ^(27,332)^ = 55.038) *p* ≤ 0.001) showed significant differences in strength between CI group.

For negative emotions, DC groups received the highest frequency and strength of attributions and MC received the least: strength of negative EA (mean = 8.03, SD ± 2.72, mean difference (MD) between DC and MC = 0.768, *p* ≤ 0.001), strength of spite EA (mean = 1.75, SD = 0.752, MD between DC and MC = 0.082, *p* ≤ 0.001), strength of embarrassment (mean = 2.03, SD = 0.788, MD between DC and MC = 0.132, *p* ≤ 0.001), strength of fear (mean = 2.19, SD = 0.812, MD between DC and MC = 0.292, *p* ≤ 0.001), frequency of fear (median = 0, IQR = 1, pairwise comparison between DC and MC = −588.412, *p* ≤ 0.001), frequency of sadness (median = 0, IQR = 0, 90th percentile = 1, pairwise comparison between DC and MC = −449.776, *p* ≤ 0.001), strength of sadness (median = 2, IQR = 1.34, pairwise comparison between DC and MC = −707.513, *p* ≤ 0.001), and frequency of embarrassment (median = 0, IQR = 0, pairwise comparison between DC and MC = 222.414, *p* ≤ 0.001).

### 3.3. Impacts of CI Group of Respondents’ Dog on EA to Their Dog

As with dog images, respondents overall attributed fewer negative emotions than positive emotions to their own dogs, as follows: sadness (*n* = 1963, 95%), fear (*n* = 2023, 98%), embarrassment (*n* = 964, 47%), spite (*n* = 517, 25%), joy (*n* = 2049, 99%), affection (2051, 99%), empathy (*n* = 1496, 73%) and compassion (*n* = 1522, 74%). Of the secondary emotions, owners attributed those with positive valence more often than those with negative valence.

No significant differences were found among CI groups in the frequency of respondents’ EA to their own dogs (one-way ANOVA, F ^(21,171)^ = 1.308, *p* = 0.398), the total frequency of secondary EA to their dog (one-way ANOVA, F ^(21,171)^ = 1306, *p* = 0.727), or of positive (one-way ANOVA, F ^(21,171)^ = 2.951, *p* = 0.122) or negative emotions (one-way ANOVA, F ^(21,172)^ = 0.422, *p* = 0.43). The only individual emotion that was attributed significantly differently across CI groups was empathy (X^2^ = 9.059, *p* = 0.030). Owners of BC dogs attributed empathy significantly less often to their dogs (80/135, 59%) than DC (437/614, 71%) and MC (308/425, 72%) dog owners.

### 3.4. Impact of CI Group of Respondents’ Dog on EA to Still Images

No significant associations were found between a respondent’s own dog’s CI group and the total number of grouped EA to different CI groups among the dog images, apart from the frequency of negative EA to DC dogs (ISKW, X^2^ = 11.091, *p* = 0.012), where DC dogs received significantly more (median = 0, IQR = 2) negative EA from BC dog owners than from MC owners (median = 0, IQR = 1) and DC owners (median = 0, IQR = 1) (*p* = 0.012).

### 3.5. LAPS Score

In the current study, the LAPS scale had an internal consistency result of alpha 0.93. Using Spearman’s rank correlation coefficient analyses, results showed that total LAPS scores were strongly positively correlated with almost all frequencies and strengths of grouped emotions at a significance of *p* < 0.001. The only exception was the frequency of negative EA to dog images, which was not significantly correlated with LAPS scores (*p* = 0.426). LAPS scores were not significantly different among CI groups of the owned dogs (ISKW, X^2^ = −0.030, *p* = 0.426).

### 3.6. C-BARQ Trainability Score

A weak, positive correlation was identified between C-BARQ trainability total scores and the frequency of EA to a respondent’s own dog (r_s_ = 0.122, *p* ≤ 0.001), and the number of secondary EA to their own dog (r_s_ = 0.081, *p* = 0.002). Trainability was also weakly significantly negatively correlated with the estimated CI group of owned dogs (r_s_ = −0.185, *p* ≤ 0.001). Mean C-BARQ trainability scores were highest for DC dogs (mean = 27.58, SD = 6.335), lower for MC dogs (mean = 25.59, SD = 5.965) and lowest for BC dogs (mean = 25.08, SD = 6.014). Significant differences were present between MC-DC (*p* ≤ 0.001) and BC-DC (*p* ≤ 0.001), but not between MC-BC (*p* = 1.000) groups.

### 3.7. TIPI Subscales

Significant weak positive correlations were found among the frequency of grouped EA to all dog images, and the openness to experience (r_s_ = 0.062, *p* = 0.007) and agreeableness (r_s_ = 0.068, *p* = 0.007) traits of respondents. Significant, weak positive correlations were also observed between the frequency of secondary EA to all dog images, and openness to experience (r_s_ = 0.049, *p* = 0.025) and agreeableness (r_s_ = 0.062, *p* = 0.007) traits. Significant negative correlations were observed between the strength of secondary EA to dog images and the emotional stability subscale (r_s_ = −0.061, *p* = 0.009). Weak but significant positive correlations were found between TIPI subscale of openness to experience, and the frequency of EA to one’s own dog (r_s_ = 0.055, *p* = 0.033). A weak negative correlation was identified between the emotional stability trait, and the frequency of secondary EA to a respondent’s own dog. Negative correlations were identified between the frequency of negative EA to a respondent’s own dog and conscientiousness (r_s_ = −0.058, *p* = 0.023) and ES (r_s_ = −0.066, *p* = 0.009). A weak positive correlation was identified between openness to experience and frequency of negative EA to the respondent’s own dog (r_s_ = 0.055, *p* = 0.034). Inversely, a weak correlation was found between agreeableness and positive emotion EA to one’s own dog (r_s_ = 0.074, *p* = 0.004) (Table 2).

## 4. Discussion

Recent studies have demonstrated that human perceptions of canine personality and preferences are influenced by canine appearance [26,46]. However, to our knowledge, the current study is the first to investigate links between canine CI and EA to dogs. This work has revealed that CI group is associated with EA to still images of dogs, with MC dog images receiving most EA overall, and more attributions of positive emotions and fewer attributions of negative emotions than DC and BC images. DC images received significantly stronger and a higher frequency of negative EA than BC and MC groups. Secondary EA was not associated with CI group for either still images of dogs or owned dogs.

The current results were unexpected for several reasons. It has been suggested that some dog–owner relationships are driven by biological mechanisms promoting caregiving to human infants [24,47,48] and that humans often view canine emotions similar to our own [49]. This may imply that secondary emotions would be attributed at a higher rate to dogs that activate the BSE systems. Given that features of dogs with high CI scores correlate with elements of BSE [50], and that dogs rating highly on BSE-associated “cuteness” were viewed most positively [26], BC dogs in the current study were expected to receive the most positive and overall EA. Our findings did not support this expectation, but they align with other reports in the literature. Hecht and Horowitz [46] systematically altered photographs of dogs to show that some BSE elements do not result in stronger self-rated aesthetic preferences from observers. In that study, two features that are more common in BC dogs than other CI groups (a rounded head and large jowls) were not associated with aesthetic preference. As such, CI score may not be consistently correlated with cuteness and aesthetic preference in all people. Other studies of companion animal species found MC conformations were preferred over BC and DC head shapes. Farnworth et al. [36] found that respondents expressed the highest preferences for MC cat faces, which may be influenced by respondents’ ownership of or familiarity with MC cats. This is supported by evidence that familiarity with a species leads to more emotions attributed to them by the individual [5].

Although the assumption that familiarity with a head shape category is linked to a preference, and perhaps an emotional attribution pattern toward similar animals seems logical, the current study demonstrates that ownership of a dog from a specific CI group was not generally linked to differences in how the respondent attributed emotions across images of different CI groups. It is therefore unlikely that more positive EA to the MC CI group derives primarily from familiarity. Rather than CI alone, pre-existing perceptions of the breeds used in the current study may have affected EA. However, this is unlikely to explain the current results fully, because the Staffordshire Bull Terrier, which in this study was represented in two of the three MC dog images, has suffered negative press in the U.K. [51] even though current MC images were rated the most positively, implying breed perception did not significantly influence EA, or that these views are variable in the public [52]. In 2013, McGreevy et al. [30] reported that eight undesirable behavioural traits correlated with CI, half of which correlated positively and half negatively. Though MC dogs may be less likely to exhibit these undesirable traits than BC and DC dogs, and this may influence respondents’ perceptions of MC dogs’ emotional capacity, trainability was negatively correlated with CI in the current study. This contrasts with Helton’s [53] findings that MC dogs were considered more trainable than other CI groups. Helton suggested BC and DC morphologies were suited to specific tasks and that this anthropogenic specialisation may influence the belief that MC dogs have a wider range of abilities. That said, such a prospect does not align with results of the current study, in that trainability was correlated negatively with CI and positively with overall frequency of EA. This may reflect differences in methodology, e.g., Helton [53] investigated breed groups rather than individual owned dogs.

As illustrated by similar studies, the current results show that primary emotions were attributed more frequently to images of dogs and respondents’ own dogs than secondary emotions were [4,5,49,54]. This aligns with the scientific consensus that primary emotions function in all invertebrates [9]. However, despite little evidence suggesting non-primate species experience secondary emotions [11], the current respondents frequently assigned secondary emotions to dog images (76% of respondents attributed at least one secondary emotion to images) and their own dogs (100% of respondents attributed at least one secondary emotion to their dog). This finding may have been influenced by a skew in respondent experience toward dog owners (84% of the overall sample) and those working professionally with dogs (22%), who have likely spent more time around dogs than other respondents, which may impact their EA to the still images presented here. That said, these attributions were on a significantly smaller scale than primary emotions (98% of respondents attributed at least one primary emotion to dog images, and 100% of respondents attributed at least two primary emotions to their own dog). This replicates results from other EA studies [2,4,54], confirming widespread belief in the U.K. that dogs experience secondary emotions.

The finding that owned dogs were attributed more emotions than images lends support to the theory that when only visual aspects of a dog are available, these carry more weight for EA considerations than when observers have other indicators available to them, such as behavioural observations. Buckland et al. [55] found that dog behaviours as well as facial expressions were used in emotional states identification. Additionally, other fundamental elements of human–animal interaction, such as attachment, may contribute to EA, potentially becoming a stronger predictor than appearance alone after a prolonged time spent with a dog through ownership [49,54].

No significant differences in the number of overall emotions or grouped primary and secondary EA were observed among CI groups of respondents’ dogs. Although still images influence adopters’ initial interest in shelter dogs [56], additional factors are considered before successful adoption [57]. Thus, it is likely that after observing factors such as behaviour and emergent bonds with a dog, CI becomes a weaker predictor of EA. However, BC dogs were attributed empathy significantly less often by their owners than DC and MC dogs. This contrasts with respondents’ pattern of EA to images and fails to explain the increased popularity of BC breeds in recent years [31], suggesting that the appeal of BC dogs may not run as deep as the attribution of secondary emotions such as empathy. McGreevy et al. [30] found a higher incidence of behaviour problems such as dog-directed aggression in BC dogs versus other groups, which may lead to owners attributing them fewer positive EA such as empathy. This finding may be reflected in the lower trainability reported for BC than for MC and DC breeds in this study. That said, it is important to note that in the current study, there were relatively fewer BC-owning respondents than MC- and DC-owning respondents, so this finding may not be generalisable to a wider population.

A novel finding in the current study was that, in contrast to negative emotions, positive emotions were more frequently attributed to both owned dogs and those in images. Of negative EA, spite frequency was not affected by CI group, though DC dogs received the strongest spite attributions compared to other groups. As the current study found a low rate of spite attributions, this may have skewed results to the characteristics of the small group of owners attributing this emotion. However, 25% of the current sample of U.K. owners reported spite in their dogs, the same as New et al. [8] investigating emotion attribution to dogs in the United States. This suggests that spite may be attributed to owned dogs similarly in Western nations.

The strength of attachment to owned dogs was positively correlated with EA frequency to both their own dog and still images. Su et al. [54] suggests that through ownership and the development of a strong bond, the owner’s brain mechanisms may be primed to attribute more emotion. The current results corroborate similar findings in the literature that attachment is directly correlated with emotion attribution frequency [54,58].

In the current study, owner personality was related to EA. Conscientiousness and emotional stability were negatively correlated with negative EA frequency to respondents’ dogs, and agreeableness was positively correlated with positive EA frequency to owned dogs and images. In a previous study, Dodman et al. [59] identified negative correlations between use of positive punishment and owner agreeableness, conscientiousness and emotional stability. These associations present a potential mechanism for the propensity to use aversive training techniques with dogs. It is possible that owners with low conscientiousness, emotional stability and agreeableness see their dogs’ emotions in a more negative light, which may lead to an increased use of positive punishment. Whether owners with these personality traits are more likely to own dogs with undesirable behaviours, inciting the use of positive punishment, or vice versa, is unclear. The prospect that the current findings may imply EA as a potential risk factor for the use of training techniques linked to poor canine welfare [17] merits further investigation to identify dogs at risk due to their owner’s beliefs. This confirms the need for the nascent science of dogmanship [60] to pay as much attention to human personality as it does to dog behaviour [61].

The current study has some limitations. Although the sample size for this study (*n* = 2445) was relatively large, the methods of sampling were not optimal for generalisability of the findings, with some unavoidable biases potentially introduced by online sampling [62]. Additionally, few of the current participants were male (9%), though this female bias replicates demographic imbalances reported in similar studies [4], so the current results are likely comparable with those in the existing companion animal literature. Dog-owning respondents completed a significantly longer survey than non-owners due to additional questions on attachment [54] and dog behaviour, which may have contributed to survey fatigue and bias in answering later questions [63]. However, owners were informed of survey length prior to completion and thus should have been prepared for its length, and simple, reliable scales such as the trainability subscale of the CBARQ were included opposed to the entire questionnaire, as they were not the focal research topic [64]. Future research should explore wider behavioural traits and their relationship with EA in pet dogs.

The current study explored eight emotions to ensure inclusion of a balance between positively and negatively valenced emotions, and both primary and secondary emotions. Other emotion motivation systems have been described in companion animals [65]. The use of only eight emotions in this study was designed to minimise survey length and respondent fatigue. However, further research into EA of facial expressions should explore other emotions relevant to human interpretation of canine behaviour, such as frustration [66].

Data on dog breeds’ average CI score were derived from Australian dogs [30]. In that study, as live dogs were used, soft tissues on the head (notably the masseter muscles) may have affected indices of more muscular breeds and compromised the validity of such scores as an absolute reflection of skull morphology. However, because this were true for all dogs measured, the live dog data likely accurately represent fundamental morphological differences across breeds. For the current study, the CI score range in McGreevy et al.’s [30] study was split into terciles to allow for the creation of CI group categories described in the Methods section of this paper. Such designations have been described as arbitrary because the CI scale has no natural breaks [67], and thus, grouped categorisations of breeds may be misleading. However, the difference between dogs on extreme ends of the CI index remains distinct and consistent, so this categorisation remains a useful tool for the purposes of the current study. As CI data for the current study were collected from Australian dogs registered with the Australian National Kennel Club (ANKC), and images used in the study presented U.K. dogs with unverifiable breed designations, it is possible that estimated CI scores were inaccurate for the dogs in the images used. That said, most ANKC breed standards were derived directly from The Kennel Club (U.K.), so marked morphological divergence between pedigree dogs in the two countries is unlikely, and it is unlikely that differences in CI scores are larger between U.K. and ANKC breeds than within breeds. It is also important to note that as breed designations were allocated by the rescue staff, they may have been indicative rather than definitive, and some dogs may have been cross breeds.

The number of images and thus breeds available for use in the current study were limited, because images needed to conform to the matched features for use in the study as well as being listed as a breed that was present in the secondary dataset from McGreevy et al. [30]. This may limit the generalisability of the current results to other breeds. Matched features may have affected EA, not least because Hecht and Horowitz [46] reported that respondents preferred canine “approximations of a smile”, and Buckland et al. [55] found participants believed an open mouth and pricked ears signified canine happiness. In the current study, both mouth shape and ear position were features that all images were kept consistent for. This may explain why, regardless of head shape depicted in the images, current participants only infrequently attributed negative valence emotions to dog images, even though negative emotions were represented in higher frequencies when attributed to owned dogs. However, given that matched features were consistent across images, it is unlikely that they affected EA results. It is important to note that the source of the images used in this study was a U.K. rescue shelter using images to raise the profile of dogs that needed rehoming; thus, it is likely that facial expressions perceived negative emotional states were avoided. Recent research [68] has highlighted that head-tilting in dogs may be linked to the processing of information important to the dog–human relationship. It is thus possible that humans may attribute emotions differently to dogs exhibiting this visual signal, which was marked in one MC image in this study, and thus may have influenced more positive EA for the MC group overall. Finally, presentation of still images were not in a randomised order, which may have introduced an order effect into responses.

## 5. Conclusions

In still images, dogs with moderate mesocephalic morphologies were regarded to experience more positive emotions than those in other skull morphology categories, with longer-muzzled dolichocephalic dogs considered to experience the most negative emotions of all CI groups. Conversely, attributions of emotion to one’s own dog was unrelated to their dog’s CI, or to the attribution of emotion to still images of other dogs. This suggests EA is influenced more by the skull morphology of unknown dogs in still images than where an owner has a relationship with an individual dog. This is likely due to owners having access to a wealth of other information on their own dog’s emotional states, such as vocalisations, behaviour and body language in a variety of contexts to inform their emotional attributions. The current results do not support the hypothesis that CI is correlated with emotional attribution, with higher CI (more brachycephalic) groups not being attributed the richest emotional lives, as predicted.

The results of the current study have several applied implications and may stimulate further research in this area. It is critical to identify whether EA has meaningful impacts on canine welfare—for example, whether there are links between EA and the use of aversive training techniques by dog owners. Where erroneous EA may be related to undesirable owner behaviour, the development of educational materials that aid owners in correctly attributing emotions may benefit canine welfare. In addition, results regarding assessment of still images of dogs are of potential use to rehoming organisations, which often rely on still images to advertise dogs online to prospective owners and would benefit from data on which aspects of appearance people consider positively.

## Figures and Tables

**Table 1:** Categorisation of individual emotions included within.

	Positive Valence	Negative Valence
Primary Emotions	Joy, Affection	Fear, Sadness
Secondary Emotions	Compassion, Empathy	Embarrassment, Spite

**Table 2:** Spearman’s rank correlation coefficient analyses between TIPI subscale scores and emotional attribution to dog images and owned dogs summary of significant associations.

Variable	TIPI Element	Correlation Coefficient	*p* Value
Frequency of positive emotions attributed to dog images	Agreeableness	0.074	<0.001
	Openness to experience	−0.066	0.004
Frequency of positive emotions attributed to own dog	Agreeableness	0.074	0.004
Frequency of negative emotions attributed to own dog	Conscientiousness	−0.058	0.023
	Emotional stability	−0.066	0.009
	Openness to experience	0.055	0.034

## Data Availability

Data is not available due to ethical and privacy agreements with participants outlining data would be viewed only by the researcher(s).
